# The effects of folic acid supplementation on endothelial function in adults: a systematic review and dose-response meta-analysis of randomized controlled trials

**DOI:** 10.1186/s12937-023-00843-y

**Published:** 2023-02-24

**Authors:** Mohammad Zamani, Fatemeh Rezaiian, Saeede Saadati, Kaveh Naseri, Damoon Ashtary-Larky, Mohsen Yousefi, Elnaz Golalipour, Cain C. T. Clark, Samira Rastgoo, Omid Asbaghi

**Affiliations:** 1 Department of Clinical Nutrition, School of Nutritional Sciences and Dietetics, Tehran University of Medical Sciences, Tehran, Iran; 2 National Nutrition and Food Technology Research Institute, Faculty of Nutrition Sciences and Food Technology, Shahid Beheshti University of Medical Sciences, Tehran, Iran; 3 Department of Medicine, School of Clinical Sciences, Monash University, Melbourne, Australia; 4 Gastroenterology and Liver Diseases Research Center, Research Institute for Gastroenterology and Liver Diseases, Shahid Beheshti University of Medical Sciences, Tehran, Iran; 5 Nutrition and Metabolic Diseases Research Center, Ahvaz Jundishapur University of Medical Sciences, Ahvaz, Iran; 6 Faculty of Medicine, Shahid Beheshti University of Medical sciences, Tehran, Iran; 7 Centre for Intelligent Healthcare, Coventry University, Coventry, CV1 5FB UK; 8 Student Research Committee, Shahid Beheshti University of Medical Sciences, Tehran, Iran; 9 Department of Cellular and Molecular Nutrition, National Nutrition and Food Technology Research Institute, Faculty of Nutrition Science and Food Technology, Shahid Beheshti University of Medical Sciences, Tehran, Iran; 10 Cancer Research Center, Shahid Beheshti University of Medical Sciences, Tehran, Iran

**Keywords:** Endothelial function, Flow mediated dilation, FMD, Folic acid supplementation, Meta-analysis

## Abstract

**Background:**

Endothelial dysfunction serves as an early marker for the risk of cardiovascular disease (CVD); therefore, it is an attractive site of therapeutic interventions to reduce the risk of CVD. This study was conducted to investigate the effect of folic acid supplementation on endothelial function markers in randomized controlled trials (RCTs).

**Methods:**

PubMed, ISI web of science, and Scopus databases were searched up to July 2022 for detecting eligible studies. A random-effects model was used for meta-analysis, and linear Meta-regression and non-linear dose-response analysis were performed to assess whether the effect of folic acid supplementation was affected by the dose and duration of intervention. Cochrane tools were also used to assess the risk of bias in the included studies.

**Results:**

Twenty-one studies, including 2025 participants (1010 cases and 1015 controls), were included in the present meta-analysis. Folic acid supplementation significantly affected the percentage of flow-mediated dilation (FMD%) (WMD: 2.59%; 95% CI: 1.51, 3.67; *P* < 0.001) and flow-mediated dilation (FMD) (WMD: 24.38 μm; 95% CI: 3.08, 45.68; *P* = 0.025), but not end-diastolic diameter (EDD) (WMD: 0.21 mm; 95% CI: − 0.09, 0.52; *P* = 0.176), and intercellular adhesion molecule (ICAM) (WMD: 0.18 ng/ml; 95% CI: − 10.02, 13.81; *P* = 0.755).

**Conclusions:**

These findings suggest that folic acid supplementation may improve endothelial function by increasing FMD and FMD% levels.

**Trial registration:**

PROSPERO registration cod: CRD42021289744.

**Supplementary Information:**

The online version contains supplementary material available at 10.1186/s12937-023-00843-y.

## Background

The importance of healthy endothelium has been increasingly recognized in the maintenance of normal vascular function [1]. Endothelial cells have a notable role in the preservation of vascular integrity, preventing platelet aggregation, regulating thrombosis, and angiogenesis through releasing of different signaling molecules [2, 3]. However, vascular problems resulting from conditions like angioplasty, stenting, diabetes, hypertension, and imbalances in the production of vasodilator molecules (nitric oxide (NO)) and vasoconstrictor substances (endothelin), can lead to endothelial dysfunction [4–6]. Loss of normal endothelial functions (endothelial dysfunction) plays a pivotal role in the progression of atherosclerosis and coronary artery disease (CAD) and it can be accompanied by various cardiovascular risk factors [7–9]. Consequently, maintaining a healthy endothelium could represent a promising therapeutic approach for the prevention of these pathological conditions [10]. Studies have suggested that supplements with endothelium-protective properties might be beneficial in the management of cardiovascular diseases [11].

Endothelial dysfunction is a systemic disorder and a key variable in the pathogenesis of atherosclerosis and its complications [12]. Several biological markers, including intercellular adhesion molecule (ICAM), flow-mediated dilation (FMD), and end-diastolic diameter (EDD), have been used as indicators of endothelial dysfunction. A systemic increase in the expression of adhesion molecules (e.g., ICAM) on the surface of endothelial cells can increase the risk of endothelium dysfunction [12]. Soluble forms of these molecules in the blood increase, which can be assessed by laboratory tests using blood serum. FMD of the brachial artery, an index of endothelium-dependent vasodilation, reflects NO production in the endothelium and correlates with coronary artery endothelial function [13]. EDD is commonly used to determine endothelium dysfunction in vascular tone modulation [14], which is dependent on a multitude of factors, ranging from NO production, prostanoids, endothelin-1, and other endothelium-derived hyperpolarizing factors [15].

Epidemiological studies have demonstrated a reduction in cardiovascular risk within societies with folic acid-fortified foods [16, 17]. Folic acid (an oxidized form of folate with high bioavailability) insufficiency has been associated with endothelial dysfunction and increased incidence of cardiovascular diseases (CVD)s [18]. Several promising findings suggest that folic acid effectively reduces atherogenesis by improving oxidative stress, inflammation, blood pressure, lipid profile, and glycemic control [19–23]. Homocysteine may be responsible for vascular endothelial cell dysfunction that occurs at the onset CVD pathology [24]. However, the underlying mechanism of action of folic acid has not been fully established. Decreased folate concentrations and/or homocysteine in high doses can increase inflammation [25]. In endothelial cells, inflammatory cytokines result in increased circulating levels of intercellular adhesion molecule (ICAM) and vascular cell adhesion molecule (VCAM); these are transmembrane proteins that promote endothelial dysfunction [8]. Folic acid reduces homocysteine levels through its anti-inflammatory characteristics with a decline in the expression of ICAM-1 [25, 26]. In addition, another important indicator of endothelial dysfunction that can be addressed is flow-mediated dilation (FMD) [27, 28]. A substantial body of evidence suggests that significant improvement in FMD is evident after folic acid supplementation [29–32], while others do not support these findings [33–35]. Furthermore, there is conflicting and uncertain evidence regarding the effectiveness of folic acid on ICAM levels [31, 36].

Based on the above considerations, and also to address the inconsistency in the literature, we conducted this comprehensive systematic review, dose-response, meta-regression, and meta-analysis study to investigate the effectiveness of folic acid supplementation on biomarkers of endothelial function in adults.

## Methods

This study was performed in line with the preferred reporting items for systematic reviews and meta-analyses (PRISMA) protocol for reporting systematic reviews and meta-analyses [37]. Moreover, we designed this meta-analysis based on PICOS criteria (Population: adults, Intervention: folic acid, Comparison: control group, Outcome: endothelial function parameters, Study: clinical trials).

### Search strategy

We systematically reviewed electronic databases including PubMed/Medline, Scopus, and ISI Web of Science to find relevant RCTs up to July 2022. The MESH and non-MESH terms were used as follows: ((“folate” OR “folic acid” OR “Vitamin M” OR “Vitamin B9” OR “Folacin” OR “Folvite” OR “Pteroylglutamic Acid” OR “folates” OR “tetrahydrofolates” OR “Formyltetrahydrofolates”) AND (“Endothelium function” OR “FMD” OR “endothelin” OR “end-diastolic diameter” OR “EDD” OR “intercellular adhesion molecule” OR “ICAM”) AND (intervention OR “controlled trial” OR randomized OR random OR randomly OR placebo OR “clinical trial” OR trial OR “randomized clinical trial” OR RCT OR trial OR trials “Cross-Over Studies” OR “Cross-Over” OR “Cross-Over Study” OR parallel OR “parallel study” OR “parallel trial”) (Supplementary Table 1). No restriction was made on the year of publication or language of the identified papers. We conducted a manual search in google scholar and the reference lists of the related publications to avoid the possibility of missing any eligible studies. Unpublished records were also not considered.

### Study selection and eligibility criteria

All recorded articles found by electronic or manual searches were exported into EndNote software for screening (EndNote X8, Thomson Reuters, New York). The title and abstract of all publications found in the initial search were evaluated independently by two investigators (D.A.L. and B.N.). To select eligible articles, the following criteria were considered: a) the population (adults aged ≥18 years); b) RCTs with either parallel or crossover design investigating the effects of folic acid supplementation on endothelial function; c) studies that reported means and standard deviations (SDs) for indicators of endothelial function (EDD, FMD%, FMD, and ICAM) or any other effect sizes, by which the calculation of means±SDs is possible; d) studies that were placebo-controlled; e) intervention was tried for 1 week or longer durations. For multiple papers from the same dataset, the most complete ones are selected. Clinical trials with an additional arm were considered two separate studies. Articles were excluded if they: a) employed children, adolescent, or pregnant women; b) were letters, comments, short communications, reviews, meta-analyses, ecologic studies, and experimental studies; c) had no control group; d) examined the impact of acid folic in combination with other ingredients where the independent effect of folic acid could not be determined.

### Data extraction

Two independent reviewers (M.R.K. and S.S.) performed the study selection, and a chief researcher (O.A) was responsible for resolving any conflicts. The subsequent information was extracted from the eligible trials: first author’s name, year of publication, study location, study design (parallel or cross-over), gender, the health status of participants, study sample size, the duration of interventions, supplementation dosage, the mean age of participants, and the mean ± SD of the EDD, FMD%, FMD and ICAM levels throughout the trial for the intervention and control groups. We converted the data reported in different units for endothelial function measures to the most frequently used ones. All studies reported FMD in percentage and micrometers (μm), and also reported EDD in millimeters (mm). For ICAM we converted all units to nanograms per milliliter (ng/ml).

### Risk of bias assessment

The Cochrane scoring system was used to evaluate the quality of the included studies [38]. This tool contained seven domains including: 1) random sequence generation, 2) allocation concealment, 3) blinding of participants and personnel, 4) blinding of outcomes assessors, 5) incomplete outcome data reporting, 6) selective reporting, and 7) other sources of bias. Each domain was given a “high risk” score if RCT comprised methodological defects that may have affected the results, a “low risk” score if the defect was considered ineffectual and an “unclear risk” score if the information was not sufficient to determine the impact. If the trial had “low risk” for all domains, it was labeled as a high-quality study with a totally low risk of bias.

### Statistical analysis

For data analysis, we utilized Stata software version 14 (StataCorp, College Station, Texas). Furthermore, we used mean change and standard deviation (SD) of the EDD, FMD%, FMD, and ICAM levels to assess the pooled effect size. Effect sizes for all variables were listed as weighted mean differences (WMDs) and 95% confidence interval (CI) [39]. When the SD of the mean difference was not reported, we calculated it using the following formula: SD _change_ = square root ([SD baseline] ^2^ + [SD final] ^2^ – [2R × SD baseline × SD final]) [40]. For studies that only reported standard error of the mean (SEM), SD was obtained using the following formula: SD = SEM × √n, where “n” is the number of subjects in each group. Cochrane’s Q test (significance accepted at *P* < 0.05) and I^2^ index were used to determine heterogeneity between studies. We assessed the presence of potential sources of between-study heterogeneity through subgroup analysis based on trial duration, intervention dose, and health status. Subgroup analyses were conducted based on, the duration of intervention (8 ≥ vs. 8 < weeks), the dosage of folic acid supplement (≥5 vs. < 5 mg/day), and health status (CVD vs. no-CVD). The potential non-linear dose-response relationship between dosage and duration of folic acid supplementation was examined by fractional polynomial modeling. Meta-regression analysis was executed to evaluate the association between pooled effect size and folic acid dosage (mg/day) and duration of intervention). A sensitivity analysis was also performed to determine the effect of each trial on the pooled effect size [41]. Publication bias was assessed by the funnel plot inspection as well as Egger’s test.

#### Certainty assessment

The overall certainty of evidence across the studies was graded according to the guidelines of the GRADE (Grading of Recommendations Assessment, Development, and Evaluation) Working Group. The quality of evidence was classified into four categories, according to the corresponding evaluation criteria: high, moderate, low, and very low [42].

## Results

### Study selection

**Fig. 1:**
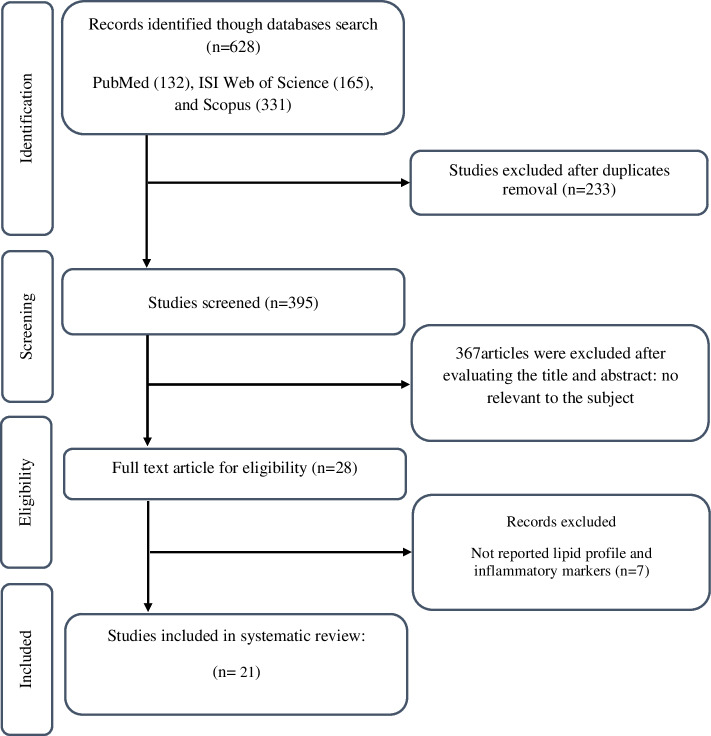
PRISMA flow diagram

The flowchart of the screening and study selection process is shown in Fig. 1. When we initiated the search, we identified a total of 628 records and recognized and removed 233 duplicates in the resultant set. Furthermore, through the title and abstract screening of 395 articles, 367 were removed. After reviewing the full texts of the remaining 28 articles, 7 trials were excluded due to not reporting the required information. Finally, 21 RCTs were included in the final meta-analysis [29–36, 43–55].

### Characteristics of the included studies

The general features of included studies are outlined in Table 1. The 21 eligible studies were published between 1999 and 2016 and had 1–52 weeks of follow-up. A total of 2025 participants were included (1010 cases and 1015 controls). These studies were carried out in the United Kingdom [33, 34, 43–48, 52], Australia [35], USA [50], China [32, 53], Iran [55], Italy [54], Greece [29], Netherland [36, 50], Canada [31] and Belgium [51]. Daily supplemental dosage of folic acid varied between 0.4 and 10 mg/day across the studies. Twelve studies had a parallel design [29, 30, 33, 34, 36, 46, 47, 49, 50, 52–55], and the rest were cross-over [31, 32, 35, 43–45, 48, 51]. All studies included both sexes [29–36, 43–53], except 2 studies that enrolled just female subjects [54, 55]. The sample size in the included trials ranged from 17 [32] to 530 [49] participants. The mean age of the individuals ranged from 26 [54] to 66 [36] years old and the mean baseline BMI varied from 24 [55] to 29 [50] kg/m^2^. Participants in these studies were patients with coronary artery disease (CAD) [30, 34, 46–48, 50, 52], type 2 diabetes mellitus (T2DM) [31, 36], acute myocardial infarction [51], hyperhomocysteinaemia [43], hypercholesterolemia [29], predialysis renal failure [33], polycystic ovary syndrome [54], preeclampsia [55], and healthy adults [32, 35, 45, 49, 53].

**Table 1:** Characteristic of included studies in meta-analysis

Studies	Country	Study Design	Participant	Sample size and Sex	Sample size		Trial Duration (Week)	Means Age		Means BMI		Intervention	
					IG	CG		IG	CG	IG	CG	Acid folic dose (mg/d)	Control group
Woo et al. 1999 [32]	China	Crossover, R, PC, DB	Healthy Adults	M/F: 17	17	17	8	54 ± 10	54 ± 10	NR	NR	10	Placebo
Bellamy et al. 1999 [43]	United Kingdom	Crossover, R, PC, DB	hyperhomocysteinaemic subjects	M/F: 18	18	18	6	NR	NR	NR	NR	5	Placebo
Title et al. 2000 [30]	United Kingdom	Paralell, R, PC, DB	Patients With Predialysis Renal Failure	M/F: 100	50	50	12	61 ± 7	62 ± 7	28.2 ± 3.2	27.5 ± 2.7	5	Placebo
Thambyrajah et al. 2000 [33]	Canada	Paralell, R, PC, DB	Coronary Artery Disease	M/F: 50	25	25	16	57.2 ± 9.8	60.6 ± 8.6	NR	NR	5	Placebo
Doshi et al. 2001 [44]	United Kingdom	Crossover, R, PC, DB	Coronary Artery Disease	M/F: 50	50	50	6	57 ± 8	57 ± 8	28.5 ± 4.4	28.5 ± 4.4	5	Placebo
Thambyrajah et al. 2001 [34]	United Kingdom	Crossover, R, PC, DB	Healthy Adults	M/F: 126	126	126	16	39 ± 12	39 ± 12	NR	NR	0.4	Placebo
Pullin et al. 2001 [45]	United Kingdom	Paralell, R, PC, DB	Coronary Artery Disease	M/F: 86	43	43	12	63 ± 4.9	63.4 ± 4.3	28.6 ± 2.8	27.2 ± 2.1	5	Placebo
Doshi et al. 2002 [46]	United Kingdom	Paralell, R, PC, DB	Coronary Artery Disease	M/F: 33	16	17	6	55 ± 7	56 ± 7	NR	NR	5	placebo
Doshi et al. 2003 [47]	United Kingdom	Paralell, R, PC, DB	Coronary Heart Disease	M/F: 83	33	50	6	56 ± 7	57 ± 8	28.9 ± 5.99	28.5 ± 4.4	5	Placebo
Doshi et al. 2004 [48]	Netherlands	Paralell, R, PC, DB	patients with type 2 diabetes mellitus and mild hyperhomocysteinaemia	M/F: 41	23	18	24	63.7 ± 8.6	66.1 ± 8.5	29.3 ± 3.9	28.8 ± 3.4	5	Placebo
Woodman et al. 2004 [35]	United Kingdom	Crossover, R, PC, DB	coronary heart disease	M/F: 50	50	50	24	57 ± 8	57 ± 8	28.5 ± 4.4	28.5 ± 4.4	5	Placebo
Lekakis et al. 2004 [29]	Greece	Paralell, R, PC, DB	patients with hypercholesterolaemia receiving statins	M/F: 34	17	17	4	55.7 ± 8.3	57.3 ± 8.8	NR	NR	5	Placebo
Spoelstra-de Man et al. 2004 [36]	Australia	Crossover, R, PC, DB	healthy hyperhomocysteinaemic subjects (High tHcy)	M/F: 26	26	26	8	49 ± 10	49 ± 10	28.1 ± 5	28.1 ± 5	5	Placebo
Durga et al. 2005 [49]	Netherlands	Paralell, R, PC, DB	men and postmenopausal women	M/F: 530	264	266	52	60 ± 5	60 ± 6	NR	NR	0.8	Placebo
Moat et al. 2006 (a) [50]	USA	Paralell, R, PC, DB	Coronary Artery Disease	M/F: 59	30	29	6	61 ± 7	61 ± 7	28.5 ± 4.4	29.6 ± 4.1	0.4	Placebo
Moat et al. 2006 (b) [50]	USA	Paralell, R, PC, DB	Coronary Artery Disease	M/F: 54	25	29	6	60 ± 7	61 ± 7	29.9 ± 4.4	29.6 ± 4.1	5	Placebo
Title et al. 2006 [31]	Canada	Crossover, R, PC, DB	type 2 diabetes	M/F: 19	19	19	2	54.5 ± 5.9	54.5 ± 5.9	NR	NR	10	Placebo
Moens et al. 2007 [51]	Belgium	Crossover, R, PC, DB	Acute Myocardial Infarction	M/F: 40	20	20	6	57 ± 11	56 ± 14	NR	NR	10	Placebo
Shirodaria et al. 2007 (a) [52]	United Kingdom	Paralell, R, PC, DB	Coronary Artery Disease	M/F: 34	20	14	1	62.2 ± 6.95	64 ± 8.9	28.2 ± 3.7	26.9 ± 4.5	0.4	Placebo
Shirodaria et al. 2007 (b) [52]	United Kingdom	Paralell, R, PC, DB	Coronary Artery Disease	M/F: 36	22	14	1	63.3 ± 7.5	64 ± 8.9	28 ± 3.5	26.9 ± 4.5	5	Placebo
Woo et al. 2008 [53]	China	Paralell, R, PC, DB	Subjects with Subnormal Intake	M/F: 104	51	53	24	45 ± 8	45 ± 8	NR	NR	5	Placebo
Palomba et al. 2010 [54]	Italy	Paralell, R, PC, DB	Polycystic Ovary Syndrome	F: 50	25	25	24	26.9 ± 3.1	26.4 ± 2.8	27.9 ± 2.6	28.1 ± 3.1	5	control group
Hashemi et al. 2016 [55]	Iran	Paralell, R, PC, TB	Pre eclamptic patients	F: 79	40	39	8	31.2 ± 4.3	30.82 ± 4.08	24.63 ± 2.64	25.19 ± 2.53	5	control group

*Abbreviations*: *IG* intervention group, *CG* control group, *DB* double-blinded, *SB* single-blinded, *PC* placebo-controlled, *CO* controlled, *RA* randomized, *NR* not reported, *F* Female, *M* Male

### Quality assessment

Based on the Cochrane risk assessment, most of the included studies had a high risk of bias [29–35, 43, 44, 46–54], however, 3 RCTs had a moderate risk of bias [36, 45, 55] (Table 2).

**Table 2:** Quality assessment

Studies	Random sequence generation	Allocation concealment	Selective reporting	Other sources of bias	Blinding (participants and personnel)	Blinding (outcome assessment)	Incomplete outcome data	Overall risk of bias
Woo et al. 1999 [32]	U	H	H	H	L	U	L	H
Bellamy et al. 1999 [43]	U	H	H	H	L	U	L	H
Thambyrajah et al. 2000 [33]	L	H	H	H	L	U	L	H
Title et al. 2000 [30]	L	H	H	H	L	U	L	H
Doshi et al. 2001 [44]	L	H	H	H	L	U	L	H
Pullin et al. 2001 [45]	L	L	H	H	L	U	L	M
Thambyrajah et al. 2001 [34]	L	H	H	H	L	U	L	H
Doshi et al. 2002 [46]	L	H	H	H	L	U	L	H
Doshi et al. 2003 [47]	L	H	H	H	L	U	L	H
Spoelstra-de Man et al. 2004 [36]	L	L	H	H	L	U	L	M
Doshi et al. 2004 [48]	L	H	H	H	L	U	L	H
Lekakis et al. 2004 [29]	L	H	H	H	L	U	L	H
Woodman et al. 2004 [35]	L	H	H	H	L	U	L	H
Durga et al. 2005 [49]	L	H	H	H	L	U	L	H
Moat et al. 2006 [50]	L	H	H	H	L	U	L	H
Title et al. 2006 [31]	L	H	H	H	L	U	L	H
Moens et al. 2007 [51]	L	H	H	H	L	U	L	H
Shirodaria et al. 2007 [52]	L	H	H	H	L	U	L	H
Woo et al. 2008 [53]	L	H	H	H	L	U	L	H
Palomba et al. 2010 [54]	L	H	H	H	L	U	L	H
Hashemi et al. 2016 [55]	L	L	H	H	L	L	L	M

Overall risk of bias: L, low-risk of bias (H < 2); M: moderate-risk of bias (H = 2); H: high-risk of bias (H > 2)

*Abreviations*: *L* low-risk of bias, *U* unclear-risk of bias, *H* high-risk of bias

### The effects of folic acid supplementation on EDD

**Fig. 2:**
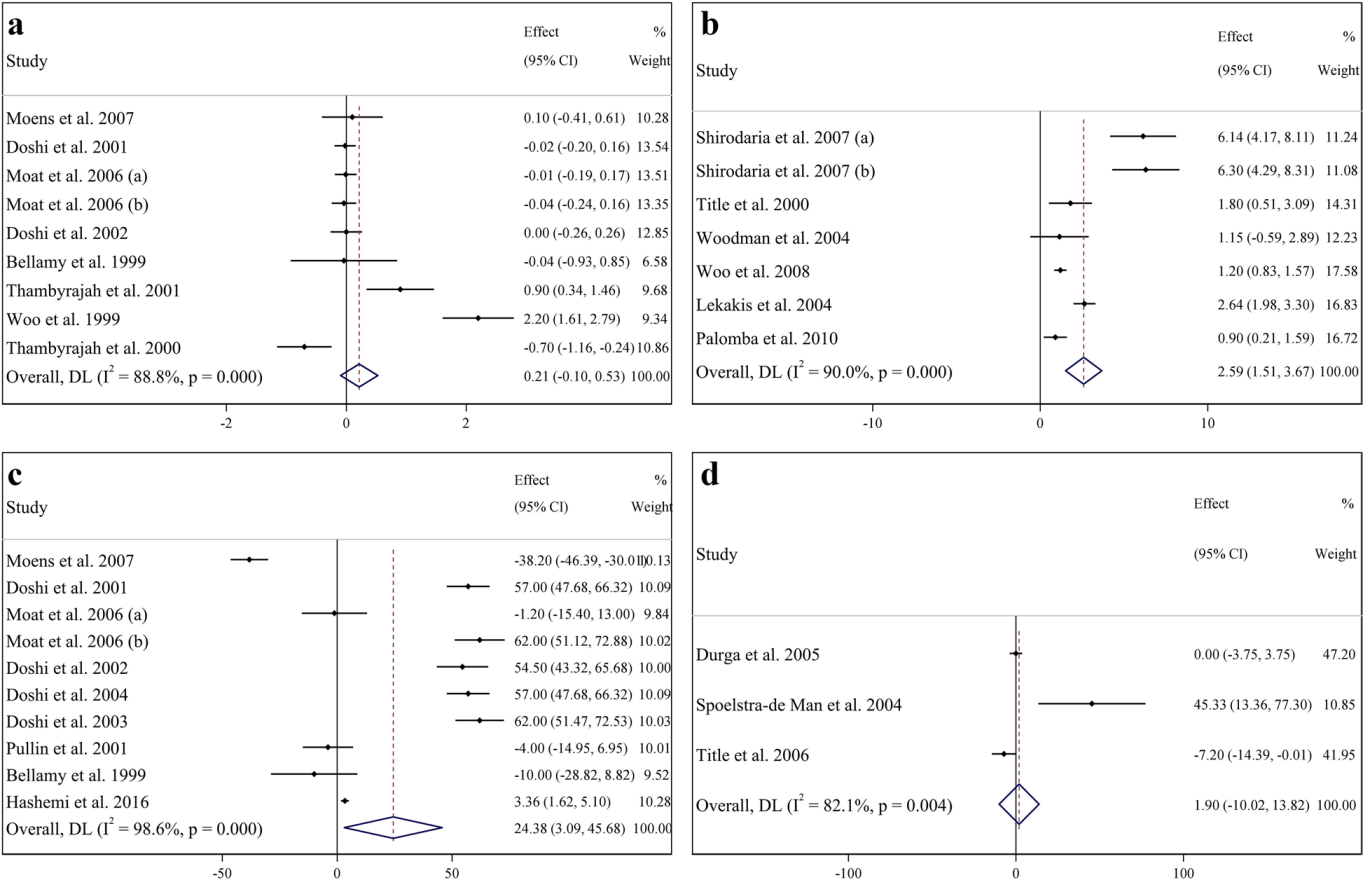
Forest plot presenting mean difference (MD) and 95% confidence intervals for the impact of folic acid supplementation on **a**) EDD (mm), **b** FMD%, (%) **c**) FMD (μm), and **d**) ICAM (ng/ml). EDD, End-Diastolic Diameter; FMD, flow mediated dilation; ICAM, Intercellular Adhesion Molecule

Findings from analysis of 9 effect sizes, including 542 subjects (269 cases and 273 controls), demonstrated that folic acid supplementation did not significantly affect the level of EDD compared to placebo (WMD: 0.21 mm; 95% CI: − 0.10, 0.53; *P* = 0.176), with significant heterogeneity among the studies (I^2^ = 88.8%, *P* < 0.001) (Fig. 2a). Moreover, subgroup analyses did not reveal significant effects (Table 3).

**Table 3:** Subgroup analyses of acid folic supplementation on endothelial function in adults

	Number of studies	WMD (95%CI)	*P*-value	heterogeneity		
				P heterogeneity	I^2^	P between sub-groups
Subgroup analyses of acid folic supplementation on EDD.						
Overall effect	9	0.21 (−0.09, 0.52)	0.176	< 0.001	88.8%	
Trial duration (week)						
< 8	6	−0.01 (−0.11, 0.08)	0.766	0.998	0.0%	0.351
≥ 8	3	0.79 (−0.90, 2.48)	0.359	< 0.001	96.7%	
Intervention dose (mg/d)						
< 5	1	−0.01 (−0.19, 0.17)	0.914	–	–	0.211
≥ 5	8	0.26 (−0.12, 0.65)	0.184	< 0.001	90.1%	
Health status						
CVD	6	0.04 (−0.10, 0.19)	0.558	0.072	50.6%	0.654
No-CVD	3	0.48 (−1.44, 2.42)	0.621	< 0.001	96.6%	
Subgroup analyses of acid folic supplementation on FMD (%).						
Overall effect	7	2.59 (1.51, 3.67)	**< 0.001**	< 0.001	90.0%	
Trial duration (week)						
< 8	3	4.90 (2.09, 7.71)	**0.001**	< 0.001	90.2%	0.010
≥ 8	4	1.17 (0.86, 1.48)	**< 0.001**	0.675	0.0%	
Intervention dose (mg/d)						
< 5	1	6.14 (4.17, 8.10)	**< 0.001**	–	–	< 0.001
≥ 5	6	2.08 (1.11, 3.05)	**< 0.001**	< 0.001	87.3%	
Health status						
CVD	3	4.67 (1.44, 7.89)	**0.004**	< 0.001	90.3%	0.063
No-CVD	4	1.51 (0.68, 2.34)	**< 0.001**	0.001	82.2%	
Subgroup analyses of acid folic supplementation on FMD (μm).						
Overall effect	10	24.38 (3.08, 45.68)	**0.025**	< 0.001	98.6%	
Trial duration (week)						
< 8	7	26.72 (−8.07, 61.53)	0.132	< 0.001	98.6%	0.745
≥ 8	3	18.72 (−14.53, 51.99)	0.270	< 0.001	98.4%	
Intervention dose (mg/d)						
< 5	2	−2.95 (−11.62, 5.71)	0.504	0.760	0.0%	0.014
≥ 5	8	31.05 (5.30, 56.80)	**0.018**	< 0.001	98.9%	
Health status						
CVD	7	36.16 (3.35, 68.98)	**0.031**	< 0.001	98.6%	0.035
No-CVD	3	0.02 (−6.88, 6.93)	0.994	0.168	43.9%	
Subgroup analyses of acid folic supplementation on ICAM.						
Overall effect	3	0.18 (−10.02, 13.81)	0.755	0.004	82.1%	

*Abbreviations*: *CI* Confidence Interval, *WMD* Weighted Mean Differences, *EDD* End-Diastolic Diameter, *FMD* flow mediated dilation, *ICAM* Intercellular Adhesion Molecule

### The effects of folic acid supplementation on FMD%

A total of 7 effect sizes, including 360 subjects (186 cases and 174 controls), evaluated the effects of folic acid supplementation on FMD%. The pooled analysis using a random-effects model indicated a significant elevation in FMD% levels following folic acid supplementation compared to placebo (WMD: 2.59%; 95% CI: 1.51, 3.67; *P* < 0.001), with a significant degree of between-study heterogeneity (I^2^ = 90.0%, *P* < 0.001) (Fig. 2b). Folic acid supplementation increased FMD% in all subgroups (Table 3).

### The effects of folic acid supplementation on FMD

Overall, 10 effect sizes, including 836 subjects (408 cases and 428 controls), evaluated the effects of folic acid supplementation on FMD levels. The pooled analysis using a random-effects model indicated a significant elevation in FMD levels following folic acid supplementation compared to placebo (WMD: 24.38 μm; 95% CI: 3.09, 45.68; *P* = 0.025), with a significant degree of between-study heterogeneity (I^2^ = 98.6%, *P* < 0.001) (Fig. 2c). Based on subgroup analysis, we found that folic acid supplementation increased FMD levels when the intervention dose was ≥5 mg/day and the intervention conducted in the CVD group (Table 3).

### The effects of folic acid supplementation on ICAM

The meta-analysis of three effect sizes involving 609 individuals (306 cases and 303 controls) revealed no significant change in ICAM levels after folic acid supplementation (WMD: 1.90 ng/ml; 95% CI: − 10.02, 13.82; *P* = 0.755), with a high heterogeneity between studies (I^2^ = 82.1%, *P* = 0.004) (Fig. 2d). Due to the limited number of studies, subgroup analysis was not possible.

### Sensitivity analysis and publication bias

**Fig. 3:**
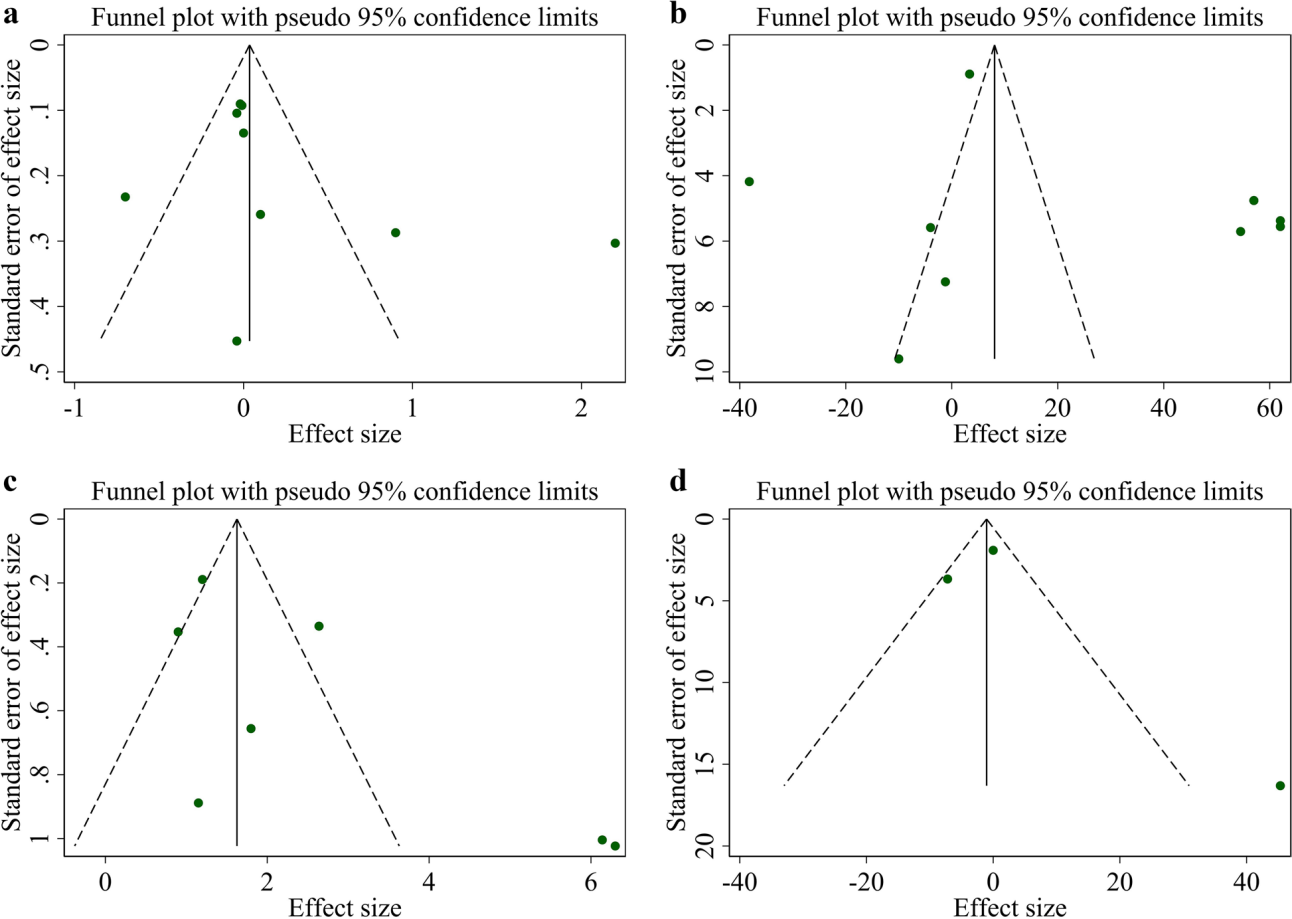
Funnel plots for the effect of folic acid supplementation on **a**) EDD (mm), **b** FMD%, (%) **c**) FMD (μm), and **d**) ICAM (ng/ml). EDD, End-Diastolic Diameter; FMD, flow mediated dilation; ICAM, Intercellular Adhesion Molecule

The sensitivity analysis suggested no significant changes following the sequential removal of each study performed for FMD%, FMD, and ICAM. However, by excluding Thambyrajah et al. study, the overall effect of folic acid supplementation on EDD was significantly changed (WMD: 0.32 mm; 95% CI: 0.00, 0.63). Furthermore, visual inspection of the funnel plot (Fig. 3a-d) and Egger’s test revealed no evidence of publication bias for studies evaluating the effects of folic acid supplementation on EDD (*P* = 0.263), FMD% (*P* = 0.100), FMD (*P* = 0.174), and ICAM (*P* = 0.642).

### Linear Meta-regression analyses between dose and duration of folic acid supplementation and endothelial function measures

**Fig. 4:**
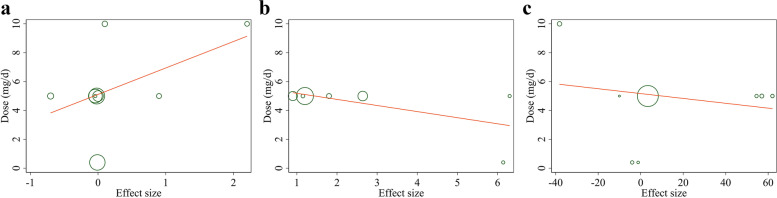
Linear meta regression plots based on dose (mg/d) of intervention for for **a**) EDD (mm), **b** FMD%, and **c**) FMD (μm). EDD, End-Diastolic Diameter; FMD, flow mediated dilation

**Fig. 5:**
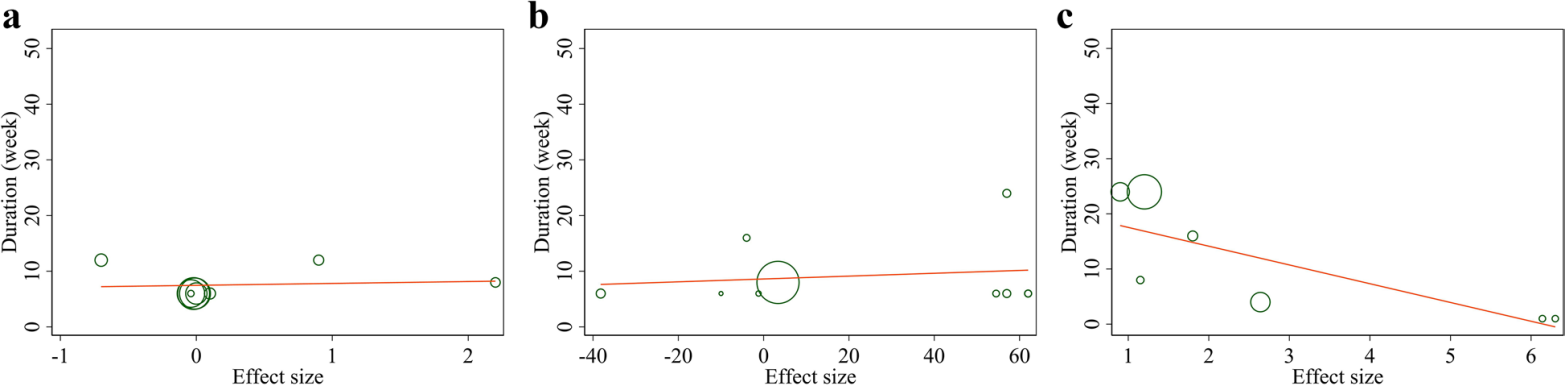
Linear meta regression plots based on duration (week) of intervention for **a**) EDD (mm), **b** FMD%, and **c** FMD (μm). EDD, End-Diastolic Diameter; FMD, flow mediated dilation

Meta-regression analysis did not show a linear relationship between dose and changes in EDD (Coefficient = 1.83, *P* = 0.15), FMD% levels (Coefficient = − 0.42, *P* = 0.13), and FMD levels (Coefficient = − 0.01, *P* = 0.68) (Fig. 4a-c). In addition, no significant relationship was found between the duration of intervention and changes in EDD (Coefficient = 0.33, *P* = 0.78) and FMD (Coefficient = 0.02, *P* = 0.66) levels (Fig. 5a, b). However, there is a significant relationship between the duration of intervention and changes in FMD% (Coefficient = − 3.40, *P* = 0.03) (Fig. 5c).

### Non-linear dose–response analyses between dose and duration of folic acid supplementation and endothelial function measures

**Fig. 6:**
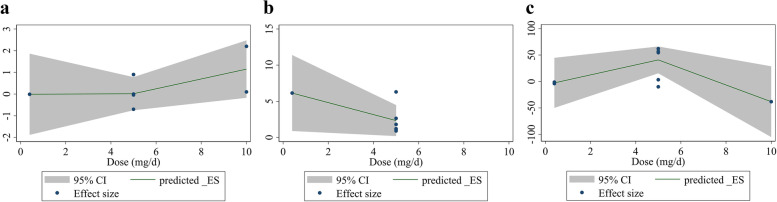
Non-linear dose-respons plots based on dose (mg/d) of intervention for **a**) EDD (mm), **b** FMD%, and **c**) FMD (μm). EDD, End-Diastolic Diameter; FMD, flow mediated dilation

**Fig. 7:**
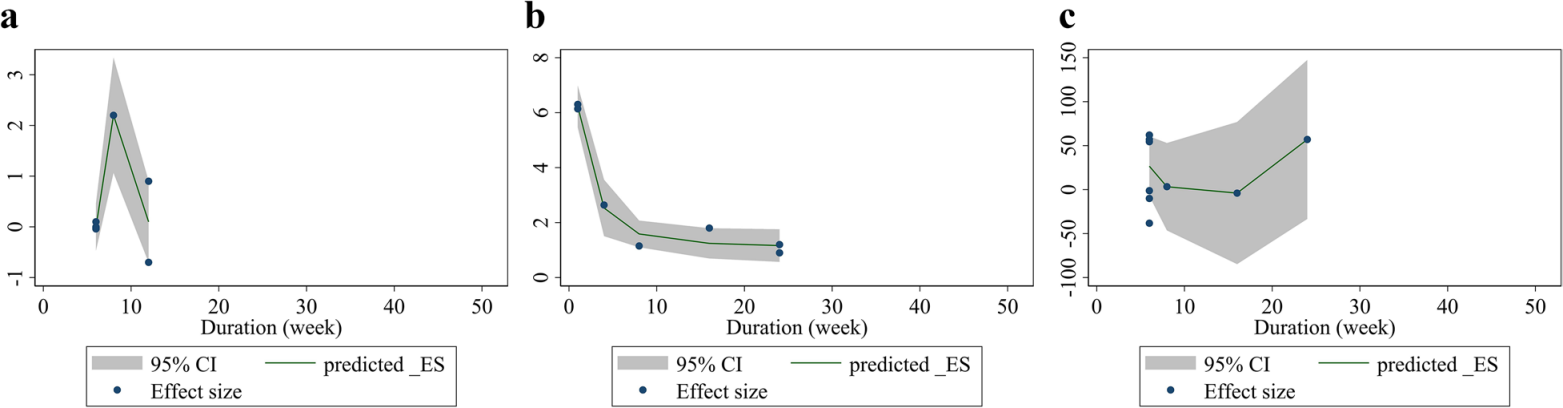
Non-linear dose-respons plots based on duration (week) of intervention for **a**) EDD (mm), **b** FMD%, and **c** FMD (μm). EDD, End-Diastolic Diameter; FMD, flow mediated dilation

Non-linear dose-response analysis demonstrated that there is a significant relationship between dose of intervention and changes in FMD (Coefficient = 1058.98, *P* = 0.035) (Fig. 6a), but not for EDD (Coefficient = − 15.24, *P* = 0.119) and FMD% (Coefficient = 0.61, *P* = 0.145) (Fig. 6b, c). Moreover, a significant relationship was shown between the duration of the intervention and changes in EDD (Coefficient = − 1989.69, *P* = 0.005) (Fig. 7a), but not for FMD% (Coefficient = 0.35, *P* = 0.051) and FMD (Coefficient = − 790.68, *P* = 0.272) (Fig. 7b, c).

#### GRADE assessment

Based on the GRADE assessment, the quality of evidence for EDD and ICAM was very low due to very serious limitations in inconsistency and serious limitations in imprecision. There was also a low quality of evidence for FMD% and FMD because of very serious limitation in inconsistency (Table 4).

**Table 4:** GRADE profile of folic acid for endothelial function parameters

Outcomes	Risk of bias	Inconsistency	Indirectness	Imprecision	Publication Bias	Quality of evidence
EDD	No serious limitation	Very serious limitation^a^	No serious limitation	Serious limitation^b^	No serious limitation	⊕◯◯◯ Very low
FMD%	No serious limitation	Very serious limitation^a^	No serious limitation	No serious limitation	No serious limitation	⊕⊕◯◯ Low
FMD	No serious limitation	Very serious limitation^a^	No serious limitation	No serious limitation	No serious limitation	⊕⊕◯◯ Low
ICAM	No serious limitation	Very serious limitation^a^	No serious limitation	Serious limitation^b^	No serious limitation	⊕◯◯◯ Very low

^a^There is high heterogeneity (I^2^ > 75%) for EDD, FMD%, FMD and ICAM

^b^There is no evidence of significant effects of folic acid supplementation on EDD, and ICAM

## Discussion

The present meta-analysis of RCTs showed that supplementation with folic acid increases FMD% compared to placebo group. In addition, high doses of folic acid (≥ 5 mg/day) and intervention in CVD patients increases FMD. However, there was no significant difference between folic acid supplementation and placebo groups regarding the levels of EDD, and ICAM.

Previous observational studies have identified an inverse link between both folic acid intake and blood folate concentration and cardiovascular health [56, 57]. Indeed, a meta-analysis involving 82,334 participants showed a 10% lower risk of stroke and a 4% lower risk of overall CVD with folic acid supplementation, especially among participants with lower plasma folate levels [58].

Folic acid exerts its protective effects against endothelial dysfunction through several mechanisms.

Folic acid improves NO bioavailability via 1) increased endothelial nitric oxide (NO) synthase (eNOS) dimerization [59] and enhancing the effectiveness of BH_4_ on eNOS uncoupling [60] and 2) serving as a direct scavenger of reactive oxygen species, which preserves bioavailable NO, both of which are mediated independent of its homocysteine-lowering effect [61].

Endothelial dysfunction, mechanistically induced by the loss of NO bioavailability [62], plays an initial role in the pathogenesis of atherosclerosis [63, 64]. Further, folate has been identified to improve endothelial dysfunction via homocysteine lowering pathways [65]. The link between elevated plasma homocysteine and endothelial cell damage has been well established and is mediated through attenuating the amount of available NO [66, 67].

Although the abovementioned mechanisms indicate both homocysteine-dependent and independent impacts of folate on endothelial function, the evidence suggests that simply lowering plasma homocysteine does not seem to improve CVD outcomes. Indeed, some studies have shown that folate supplementation with dosages ≥5 mg/day is required for its endothelial benefits, even without further decrease in homocysteine levels [31, 46, 68].

The FMD is a frequently used method to test endothelial dysfunction which indicates the bioavailability of endothelium-derived NO [62]. Our results are consistent with the findings of previous studies that demonstrated an improvement in FMD with high doses of folic acid (≥5 mg) in unhealthy subjects [46, 50]. However; some studies have reported that despite a significant reduction in total serum homocysteine, low-dose folic acid (400 μg/day) did not affect endothelial function in healthy adults [45, 49]. Doshi et al. demonstrated a significant improvement in FMD with folic acid supplementation, even before the reduction in plasma homocysteine concentration occurred, indicating that the enhancement was independent of the changes in homocysteine levels [46].

Inflammation is pivotally involved in all stages of atherosclerosis [69]. Endothelial cell dysfunction (ECD) is the earliest detectable manifestation of atherosclerotic lesions. The injured endothelium begins to increase the expression of adhesion molecules, such as E-selectin, vascular cell adhesion molecule-1 (VCAM-1), and ICAM-1, which recruits monocytes and T lymphocytes to the endothelium and leads to the inflammatory response propagation [70, 71]. Our findings from three RCTs demonstrated that supplementation with folic acid was not associated with any significant change in plasma concentrations of ICAM. However, the lack of improvement in ICAM concentrations in this study does not preclude a direct or indirect impact of folate on ICAM levels, as it might be influenced by other mediatory factors, such as blood homocysteine levels which we did not include in our data analysis. Further future large-scale studies need to be carried out on healthy populations and in subjects with reversible vascular dysfunction to better discern the effect of FA supplementation.

The present study possesses notable strengths. First, to our knowledge, this is the first systematic review and meta-analysis to investigate the effects of folic acid supplementation on a range of biomarkers of endothelial dysfunction. Another strength of this meta-analysis relates to the inclusion of several long-term studies, which has the advantage of documenting the long-term effects of folic acid supplementation on endothelial markers and allowing comparisons to shorter-duration designs. However, the existence of publication bias and heterogeneity in the analysis should be interpreted as limitations to this study.

## Conclusion

In conclusion, our data suggest that pharmacological doses of folic acid supplementation, especially in higher doses (≥ 5 mg/day), were associated with a significant improvement in endothelial function as measured with FMD and FMD%. Further in vivo mechanistic research in humans will elucidate the protective and therapeutic roles of folate in the development and progression of endothelial dysfunction in both healthy and unhealthy populations. *Finally, more research is required to* establish the safety of long-term intake before recommending it as a routine medication.

## Supplementary Information

**Additional file 1.**

## Data Availability

Not applicable.
