# Supplementary material for: The effects of folic acid supplementation on endothelial function in adults: a systematic review and dose-response meta-analysis of randomized controlled trials
Source: Nutr J. 2023 Feb 24;22:12. doi: 10.1186/s12937-023-00843-y (PMC9951414; doi:10.1186/s12937-023-00843-y)
Supplement: Supplementary file 1 — Additional file 1. [file 12937_2023_843_MOESM1_ESM.docx]

| terms | |
| --- | --- |
| "folate" OR "folic acid" OR "Vitamin M" OR "Vitamin B9" OR "Folacin" OR "Folvite" OR "Pteroylglutamic Acid" OR "folates" OR "tetrahydrofolates" OR "Formyltetrahydrofolates" | "Endothelium function" OR "FMD" OR "endothelin" OR "intercellular adhesion molecule" OR "ICAM" OR "vascular adhesion molecule" OR "VCAM" |

July 2022

| PubMed | ("folate"[Title/Abstract] OR "folic acid"[Title/Abstract] OR "Vitamin M"[Title/Abstract] OR "Vitamin B9"[Title/Abstract] OR "Folacin"[Title/Abstract] OR "Folvite"[Title/Abstract] OR "Pteroylglutamic Acid"[Title/Abstract] OR "folates"[Title/Abstract] OR "tetrahydrofolates"[Title/Abstract] OR "Formyltetrahydrofolates"[Title/Abstract])) AND ("Endothelium function"[Title/Abstract] OR "FMD"[Title/Abstract] OR "endothelin"[Title/Abstract] OR "intercellular adhesion molecule"[Title/Abstract] OR "ICAM"[Title/Abstract] OR "vascular adhesion molecule"[Title/Abstract] OR "VCAM"[Title/Abstract]) | 132 |
| --- | --- | --- |
| Scopus | ( TITLE-ABS-KEY ( "folate" OR "folic acid" OR "Vitamin M" OR "Vitamin B9" OR "Folacin" OR "Folvite" OR "Pteroylglutamic Acid" OR "folates" OR "tetrahydrofolates" OR "Formyltetrahydrofolates" ) AND TITLE-ABS-KEY ( "Endothelium function" OR "FMD" OR "endothelin" OR "intercellular adhesion molecule" OR "ICAM" OR "vascular adhesion molecule" OR "VCAM" ) ) | 331 |
| Web of science | *"folate" OR "folic acid" OR "Vitamin M" OR "Vitamin B9" OR "Folacin" OR "folvate" OR "Pteroylglutamic Acid" OR "folates" OR "tetrahydrofolate" OR "formyltetrahydrofolate" (Topic) and "Endothelium function" OR "FMD" OR "endothelin" OR "intercellular adhesion molecule" OR "ICAM" OR "vascular adhesion molecule" OR "VCAM" (Topic) \| 167 results* | 165 |
| All |  | 628 |
| duplicates |  | 233 |
| remained |  | 395 |
| included |  |  |
